# Antimicrobial use in patients with end-of-life status in intensive care units: A systematic review and meta-analysis

**DOI:** 10.20407/fmj.2025-020

**Published:** 2025-11-05

**Authors:** Yoshifumi Kubota, Akane Takamatsu, Yuya Kawamoto, Yohei Doi, Hitoshi Honda

**Affiliations:** 1 Department of Emergency and Critical Care Medicine, National Hospital Organization Nagasaki Medical Center, Omura, Nagasaki, Japan; 2 Graduate School of Public Health, St. Luke’s International University, Chuo, Tokyo, Japan; 3 Department of Infectious Diseases, Fujita Health University, School of Medicine, Toyoake, Aichi, Japan; 4 Division of Infectious Diseases, University of Pittsburgh School of Medicine, Pittsburgh, PA, USA

**Keywords:** Intensive care units, End-of-life care, Antimicrobials, Antimicrobial stewardship, Drug utilization

## Abstract

**Objectives:**

Advances in critical care have increased antimicrobial use in intensive care units (ICUs), often extending to end-of-life patients without clear clinical benefit. This systematic review and meta-analysis investigated antimicrobial use in critically ill ICU patients with end-of-life care status.

**Methods:**

A comprehensive search of Medline (PubMed) and Embase identified articles published from January 2000 through August 2023. Interventional and observational studies focusing on antimicrobial use for critically ill ICU patients with end-of-life status were included. Study types, demographics, clinical characteristics, and antimicrobial use (i.e., continuation or discontinuation) were extracted. A meta-analysis was conducted to estimate the proportion of antimicrobial use, with subgroup analyses by region and national income status.

**Results:**

From 13,542 publications, 26 studies met the inclusion criteria; no randomized or prospective studies were identified. Thirteen studies (50.0%) reported antimicrobial use and were included in the quantitative synthesis. The pooled proportion of antimicrobial prescriptions was 0.35 (95% CI, 0.18–0.54) with significant heterogeneity (I^2^=99.7%, P<0.01). Subgroup analysis revealed regional differences: 0.50 (95% CI, 0.11–0.89) in North America, 0.40 (95% CI, 0.10–0.76) in Europe, and 0.24 (95% CI, 0.10–0.76) in the Asia-Pacific region.

**Conclusions:**

Despite increasing emphasis on judicious antimicrobial use, studies comprehensively assessing antimicrobial prescribing in ICU patients with end-of-life care status remain scarce. Based on the limited available evidence, approximately one-third of such patients received antimicrobials. Regional differences in prescribing patterns were also observed, potentially influencing overall antimicrobial consumption in ICUs.

## Introduction

The field of critical care medicine has been evolving over several decades, particularly in high-income countries.^1^ Advances in intensive care management for critically ill conditions such as sepsis have improved patient outcomes while increasing the consumption of various healthcare resources, including antimicrobials, in intensive care units (ICUs).^2^ Moreover, the rising burden of infectious diseases due to multidrug-resistant bacteria has further escalated antimicrobial use. A study revealed that approximately 60% of patients admitted to ICUs in high-income countries receive antimicrobial therapy during their ICU stay.^3^ Because antimicrobials are often administered for life-saving purposes among critically ill patients in the ICU, their overuse is less frequently addressed—especially in the context of antimicrobial stewardship in current medical practice.^4^ Overuse of antimicrobials at the end of life is common, particularly among patients with advanced malignancies, even when the likelihood of recovery remains slim despite appropriate infection management. However, studies investigating antimicrobial overuse in terminally ill patients admitted to ICUs are scarce. According to guidelines for the withdrawal of life-sustaining measures in the ICU, discontinuing antimicrobials is recommended.^5^ Antimicrobial use has been linked to the emergence of drug-resistant organisms in terminally ill patients who die without withdrawal orders in the ICU.^6^ The need for end-of-life care decisions that focus on palliative measures—including the withholding of antimicrobial use among ICU patients—should be recognized. Given the uncertain status of antimicrobial use in the context of end-of-life care in ICU settings, the present systematic review and meta-analysis aimed to describe the proportion of antimicrobial use in patients receiving end-of-life care in ICUs and to explore differences in its use across geographical regions.

## Methods

### Inclusion and exclusion criteria

The study designs included in this systematic review and meta-analysis were interventional studies (e.g., randomized controlled trials, quasi-experimental studies, and before-and-after studies) and observational studies (e.g., cohort studies, case-control studies, ecological studies, and descriptive epidemiological studies). We included studies that focused on ICU patients receiving end-of-life care, specifically those describing antimicrobial use during this phase. Given the lack of a clear definition of end-of-life care status in the ICU, we defined end-of-life care in this review as care involving a withdrawal order, a withholding order, or other limitations on life-sustaining treatment. Studies primarily focused on pediatric patients, questionnaire-based investigations, case reports, conference abstracts, pooled analyses, editorials, and review articles were excluded.

### Search strategies

We conducted a comprehensive search of the Medline (PubMed) and Embase databases for articles published from January 2000 through August 2023 regarding antimicrobial use in the ICU. Given the growing focus on end-of-life care in ICUs over recent decades, the search was restricted to studies published within this time frame.^7^ The search was limited to studies published in English and involving human subjects. The search strategy for antimicrobial use in end-of-life care in the ICU was originally developed by one of the authors (A.T.), and the full search terms for both databases are provided in the Supplementary Table.

The titles of all studies identified through the search were initially screened by one reviewer (Y. Kubota) according to the inclusion criteria. Studies whose titles clearly did not meet the inclusion criteria were excluded at this stage. Following the initial screening, abstracts of potentially eligible studies were independently assessed by two reviewers (Y. Kubota and Y. Kawamoto) to select studies for comprehensive full-text evaluation. The retrieved full-text articles were then independently reviewed by the same two reviewers. In cases of disagreement, the remaining two reviewers (A.T. and H.H.) discussed the articles and made the final decision on inclusion in the systematic review and meta-analysis.

### Data extraction

We developed a data collection form for systematic data extraction using Microsoft Excel. The form included the following fields: study title, publication year, country and region, national income status, primary study objective, number of involved institutions (i.e., single-center or multicenter study), study duration, patient inclusion criteria, number of patients, patient demographics (i.e., age and sex), definition of end-of-life, outcomes related to antimicrobial use in patients with end-of-life status in the ICU, and antimicrobial use as well as discontinuation of antimicrobials among ICU patients with end-of-life status. Each national income status was determined according to the World Bank country and lending group classifications.^8^

### Quality assessment and risk of bias

The risk of bias for each study was evaluated using the Newcastle–Ottawa Scale (NOS), a tool designed to assess the quality of observational studies.^9^ The NOS for cohort studies was applied to cohort studies, and the NOS for case-control studies was used for case-control studies. Because no appropriate tool was available for assessing the risk of bias in other observational designs, including descriptive epidemiological and ecological studies, risk of bias assessment could not be performed for studies with these designs.

The NOS evaluates three domains: sample selection, comparability, and outcomes/exposure. Scores range from 0 to 4 for sample selection, 0 to 2 for comparability, and 0 to 3 for outcomes/exposure. The developers of the NOS have not provided specific guidance on interpreting risk of bias based on these scores. Following previous reports, we converted NOS scores into Agency for Healthcare Research and Quality standards using the following thresholds.^10^ Studies were classified as good quality if they received 3 or 4 stars in the selection domain, 1 or 2 stars in the comparability domain, and 2 or 3 stars in the outcome/exposure domain. Fair-quality studies were those that received 2 stars in selection, 1 or 2 stars in comparability, and 2 or 3 stars in outcomes/exposure. Studies were considered poor quality if they received 0 or 1 star in selection, or 0 stars in comparability, or 0 or 1 star in outcomes/exposure.

### Statistical analysis

The primary purpose of the meta-analysis was to assess the proportion of antimicrobial prescriptions in patients receiving end-of-life care in ICUs. Subgroup meta-analyses based on variables of interest, including study region (i.e., North America, Europe, and the Asia-Pacific region) and national income status (i.e., high- and middle-income countries), were also conducted.

The extracted data on the proportion of antimicrobial use among ICU patients receiving end-of-life care from eligible studies were pooled and analyzed using a random-effects meta-analysis model. Pooled estimates were presented as a forest plot with 95% confidence intervals (CIs). The Freeman–Tukey transformation of proportions was applied when performing the meta-analysis because the goal was to estimate a single proportion—specifically, the proportion of antimicrobial use.^11^

Heterogeneity was measured using the I^2^ index and interpreted as follows: 0%–40% may represent negligible heterogeneity, 30%–60% may represent moderate heterogeneity, 50%–90% may represent substantial heterogeneity, and 75%–100% indicates considerable heterogeneity.^12^ A funnel plot was generated to examine whether the studies included in this systematic review and meta-analysis were influenced by publication bias, which would be indicated by asymmetry in the funnel plot. All statistical analyses were performed using Stata version 18 (StataCorp, College Station, TX, USA). This systematic review and meta-analysis was conducted in accordance with the Preferred Reporting Items for Systematic Reviews and Meta-Analyses (PRISMA) 2020 guideline.^13^

## Results

**Figure 1:**
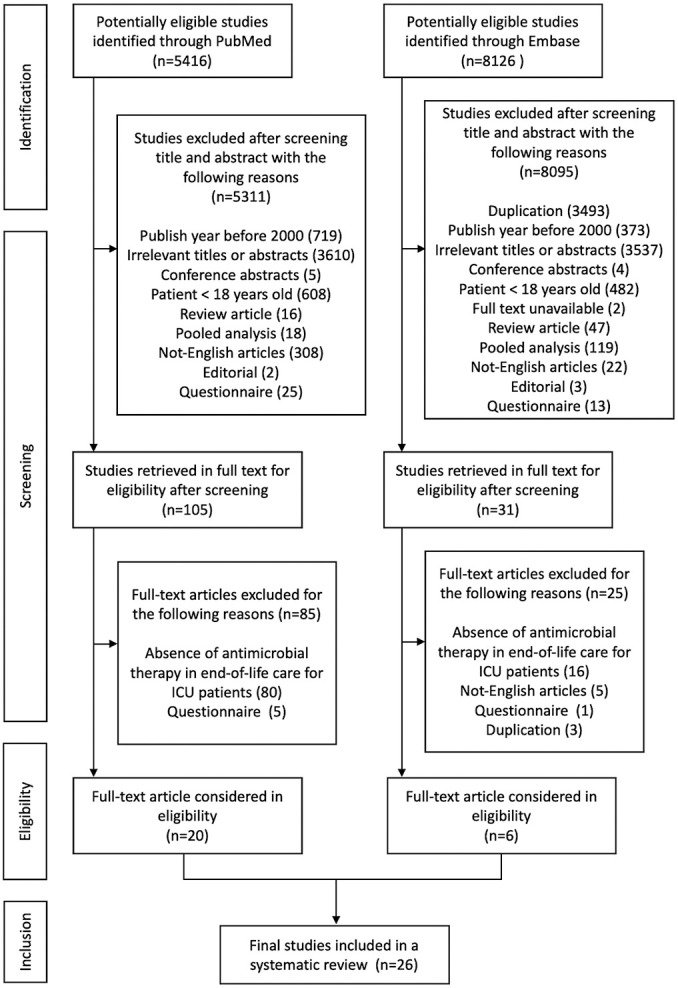
PRISMA diagram of literature search and study selection Abbreviations: ICU, intensive care unit; PRISMA, Preferred Reporting Items for Systematic Reviews and Meta-Analyses

The initial search identified a total of 13,542 publications potentially relevant to antimicrobial use in end-of-life care in the ICU (Figure 1). After excluding studies deemed ineligible based on title and abstract screening, 136 articles were retrieved for full-text review. Of these, 26 studies met the inclusion criteria and were included in the qualitative synthesis (Table 1).^14–39^ Regarding study type among the 26 included studies, 16 (61.5%) were descriptive epidemiological studies, 7 (26.9%) were cohort studies, 2 (7.7%) were ecological studies, and 1 (3.8%) was a case-control study. No interventional studies were identified. There was a gradual increase in the number of publications over time. In terms of geographic distribution, 12 studies (46.2%) were conducted in the Asia-Pacific region, 9 (34.6%) in Europe, 4 (15.4%) in North America, and 1 (3.8%) in Latin America. Based on national income status, 18 studies (69.2%) were conducted in high-income countries, while the remaining 8 (30.8%) were conducted in middle-income countries.

With respect to study objectives, only 1 of the 26 studies (3.8%) primarily focused on antimicrobial use in ICU patients with end-of-life status.^27^ This retrospective study evaluated antimicrobial use and factors associated with de-escalation in inpatients who had life-sustaining treatments withdrawn and died within 7 days, underscoring the need for careful consideration of antimicrobial stewardship in end-of-life care following the decision to suspend life-sustaining measures. The details of each study are provided in Table 1.

### Meta-analysis

**Figure 2:**
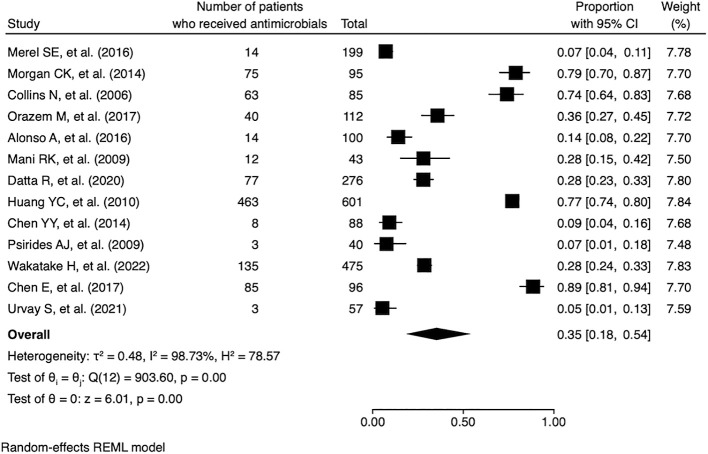
Proportion of antimicrobial use in ICU patients receiving end-of-life care Abbreviations: ICU, intensive care unit; REML, residual maximum likelihood

**Figure 3:**
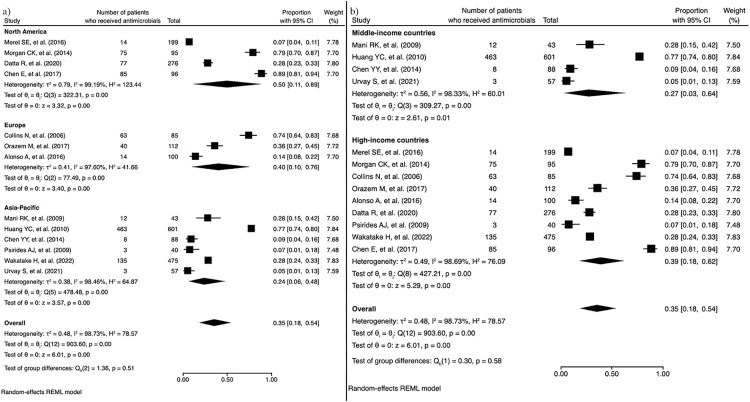
Forest plot showing antimicrobial usage rates in subgroup analyses by (a) region and (b) income status Abbreviation: REML, residual maximum likelihood

**Figure 4:**
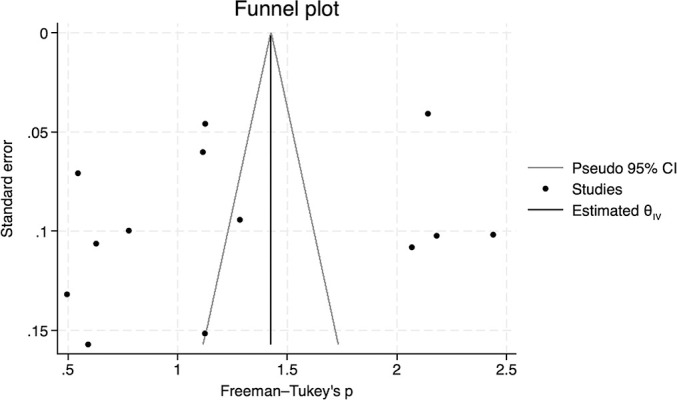
Funnel plot assessing publication bias in the included studies

Of the 26 studies, 13 (50.0%) reported the proportion of antimicrobial use in ICU patients receiving end-of-life care, and these 13 studies were included in the quantitative synthesis (Figure 2). The pooled proportion of antimicrobial use in these patients was 0.35 (95% CI, 0.18–0.54). The median I^2^ was 99.7% (P<0.01), indicating considerable heterogeneity. Subgroup meta-analyses were conducted based on geographic region and national income status (Figure 3). The proportion of antimicrobial use was 0.50 (95% CI, 0.11–0.89) in North America, 0.40 (95% CI, 0.10–0.76) in Europe, and 0.24 (95% CI, 0.10–0.76) in the Asia-Pacific region. Significant heterogeneity was observed across regions, with I^2^ values of 99.2% (P<0.01) for North America, 97.6% (P<0.01) for Europe, and 98.5% (P<0.01) for the Asia-Pacific region. In the subgroup analysis by national income status, the proportion of antimicrobial use was 0.27 (95% CI, 0.03–0.64) in middle-income countries and 0.39 (95% CI, 0.18–0.62) in high-income countries. The funnel plot assessing publication bias is shown in Figure 4. The asymmetrical distribution of studies suggested that publication bias was likely present.

### Study quality and risk of bias

The seven cohort studies were assessed using the NOS for cohort studies, while the single case-control study was evaluated using the NOS for case-control studies (Table 2). The NOS scores for the cohort studies ranged from 6 to 9, with a median score of 6. The case-control study received a NOS score of 7. Of the eight studies assessed using the NOS, only one was rated as good quality, while the remaining seven were classified as poor quality.

## Discussion

This systematic review examined antimicrobial use in critically ill patients with end-of-life status in the ICU. Given the lack of comprehensive assessment of antimicrobial use in this population, the findings of this review and meta-analysis may help raise awareness about the appropriate use of antimicrobial agents in end-of-life care—an effort we hope will contribute meaningfully to optimizing antimicrobial stewardship in this clinical setting. In this review, only 26 studies were identified, with just one study primarily focused on antimicrobial use in ICU patients with end-of-life status. This limited number of studies may reflect the ICU’s primary focus on life-sustaining treatment, which can conflict with the goals of end-of-life care. The absence of a clear definition regarding whether antimicrobial use constitutes part of end-of-life ICU care has further stalled discussion in this area. Additionally, decisions about whether to use or discontinue antimicrobials during end-of-life care are often nuanced and raise complex ethical and legal dilemmas.^40^ These challenges may hinder clinical research in this field, despite the recognized importance of judicious antimicrobial use.

In the present meta-analysis examining the proportion of antimicrobial use among ICU patients with end-of-life status, antimicrobials were administered to 35% of patients. By contrast, a previous systematic review reported that antimicrobials were used in 48 of 72 studies (66.7%) involving end-of-life non-ICU patients with advanced cancer or dementia, with the proportion exceeding 50% in this population.^41^ Although antimicrobial use appears lower in the ICU than in non-ICU settings, consumption in the ICU may still be substantial because of antimicrobial resistance and the severity of infections, which often necessitate the use of multiple broad-spectrum agents, higher doses, and prolonged courses of therapy.^42^ Moreover, excessive antimicrobial administration is associated with significant adverse events that can undermine the goals of palliative care in ICU patients at the end of life. One study revealed that antimicrobial-related adverse events occur in approximately 20% of patients, with each 10-day extension of treatment linked to a 3% increased risk of adverse events.^43^ Additionally, research has shown an association between the absence of withdrawal orders and the emergence of resistant pathogens in the ICU,^6^ raising concerns that end-of-life ICU patients receiving excessive antimicrobial therapy may face a heightened risk of acquiring multidrug-resistant organisms. Given the complexity surrounding antimicrobial use in this context, adopting the concept of a time-limited trial may help guide more optimal and judicious use of antimicrobials in end-of-life ICU care.^44,45^

One of the key findings of the present meta-analysis is that the proportion of antimicrobial use in North America (with all included studies originating from U.S. institutions) was higher than that observed in European nations and the Asia-Pacific region. This may reflect a more aggressive approach to end-of-life care in ICUs in the U.S. A questionnaire survey reported that U.S. intensivists placed greater emphasis on written decision-making than did their European counterparts.^46^ The same survey also noted that U.S.-based physicians were more likely to initiate antimicrobial therapy when end-of-life patients developed sepsis in the ICU.^46^ Moreover, a study from U.S. institutions highlighted that shared decision-making between healthcare providers and patients or family members was often incomplete for ICU patients at the end of life, largely because of the patients’ lack of decision-making capacity, which may contribute to the continuation of aggressive interventions.^47^

Unfortunately, our systematic review identified only one study that primarily focused on antimicrobial use in ICU patients with end-of-life status. The limited evidence on this topic highlights the need for further research to better understand antimicrobial use in the ICU within the context of end-of-life care. Additionally, data on the duration of ICU stay—particularly the timeframe following the transition to withdrawal or withholding of care—as well as the total days of antimicrobial therapy before and after these decisions, would be essential to more accurately visualize antimicrobial consumption among critically ill patients receiving end-of-life care.

The present study has several limitations. First, the meta-analysis revealed substantial heterogeneity across studies, primarily due to the lack of standardized study methodologies, inconsistent definitions of end-of-life status, and variations in antimicrobial practices across different regions. This is supported by previous questionnaire surveys reporting both international and intra-regional differences in antimicrobial use in end-of-life ICU care.^46,48^ Nevertheless, conducting a meta-analysis in this population and setting offers valuable insights into current antimicrobial practices and helps identify areas for improvement. Second, this meta-analysis was limited to a qualitative evaluation of the proportion of antimicrobial use in ICU patients with end-of-life status. Most studies did not provide detailed information on antimicrobial use, such as the clinical indication (treatment versus symptom relief), dosage, duration, specific agents used, or whether antimicrobials were administered based on family requests. In-depth studies focusing on prescribing practices in this context are therefore needed. Third, the risk of bias poses another limitation because many of the included studies were rated as low quality. Among the seven cohort studies and one case-control study assessed using the NOS, all but one were classified as poor quality, largely due to shortcomings in the comparability domain, including inadequate matching of exposed and non-exposed populations and insufficient adjustment for potential confounders in statistical analyses. Additionally, the risk of bias for descriptive epidemiological and ecological studies could not be assessed because of the lack of appropriate evaluation tools. Lastly, the presence of publication bias, as suggested by the funnel plot, further limits the strength of the findings. Future research efforts are warranted to clarify and improve antimicrobial practices in this population and clinical setting.

In conclusion, this systematic review and meta-analysis found that approximately one-third of ICU patients with end-of-life status received antimicrobial agents, a finding that may reflect potential overuse without clear clinical benefit. Regional differences in the proportion of antimicrobial prescribing in end-of-life ICU care were also observed. While interventions to promote appropriate antimicrobial use in this population are crucial, research specifically addressing this issue remains limited. The findings of this review highlight opportunities to reassess antimicrobial practices and underscore the need for further research to guide end-of-life care in ICUs.

## Figures and Tables

**Table 1:** Characteristics of the 26 studies included in the systematic review

Authors (Year, country, region)	Income level	Study type	Primary aim of the study	Study population (N)	EOL definition	Outcomes of antimicrobial use in EOL care in ICU	Proportion of antimicrobial use
Merel et al.^14^ (2016, USA, North America)	High	Cohort study	To examine the frequency of antimicrobial use in the acute care setting after transition to comfort-focused care and to identify patient characteristics associated with antimicrobial use.	Patients with no escalation of care in medicine, surgery, and cardiology ICUs (N=199)	Cessation of vital signs monitoring and laboratory testing, with standard orders for symptom management.	Fourteen patients continued antibiotic use after comfort care order.	7.0% (14/199)
Morgan et al.^15^ (2014, USA, North America)	High	Descriptive epidemiological study	To determine the prevalence of no-escalation-of-care designation for ICU decedents and to identify involved interventions.	Patients who died in the medical ICU (N=95)	Withholding or withdrawal of therapy before death.	Seventy-five patients continued antimicrobials, and 9 patients had antimicrobials withdrawn with no escalation of care designation.	78.9% (75/95)
Choudhuri et al.^16^ (2020, India, Asia-Pacific)	Middle	Cohort study	To compare early versus late initiation of EOL care regarding ICU mortality, length of stay, and antimicrobial consumption.	Patients identified for treatment futility in the ICU (N=107)	No initiation/escalation of LST, including antibiotics.	Antimicrobial-free days were higher in the early group (12±5.2 vs. 6±7.5 days; P=0.02).	N/A
Collins et al.^17^ (2006, Ireland, Europe)	High	Descriptive epidemiological study	To examine the incidence and pattern of limiting futile LST in an Irish ICU.	Patients who died in the ICU (N=85)	Limitation of LST prior to death.	Seventy patients continued antimicrobials, and 4 patients had antimicrobials withdrawn after EOL decision.	74.1% (63/85)
Bloomer et al.^18^ (2010, Australia, Asia-Pacific)	High	Descriptive epidemiological study	To review EOL processes and family involvement in the ICU setting.	Patients who died in mixed medical/surgical ICUs (N=34)	Treatments withheld or withdrawn.	One patient withdrew antimicrobials.	N/A
Orazem et al.^19^ (2017, Slovenia, Europe)	High	Descriptive epidemiological study	To determine how EOL decisions on limitations of LST are made in different types of Slovenian ICUs.	Patients with limitations on LST (N=112)	Limitation of LST.	Forty patients continued antimicrobials after limitation, and 24 patients withdrew antimicrobials.	35.7% (40/112)
Alonso et al.^20^ (2016, Germany, Europe)	High	Cohort study	To describe and analyze EOL decisions for patients with stroke who died in-hospital in the stroke unit.	Patients who died in the stroke care unit (N=40)	Transition from LST to withdrawal or withholding of ventilation, surgery, or circulatory support.	Antimicrobials were not ordered or were withdrawn in 86% of cases.	14.0% (34/40)
Mani et al.^21^ (2009, India, Asia-Pacific)	Middle	Cohort study	To document the EOL and full-support decisions among ICU patients and describe the decision-making process.	Patients who died in the ICU with an EOL decision (N=43)	DNR, withholding, and withdrawal of life support.	Antimicrobials were withheld in 7 patients, and antimicrobials were changed in 5 patients.	27.9% (12/43)
Graw et al.^22^ (2016, Germany, Europe)	High	Descriptive epidemiological study	To assess whether withholding blood transfusions as an initial EOL decision also applies to surgical ICU patients.	Patients who received a decision to “withhold or withdraw life support” in the surgical ICU (N=157)	Decisions to “withhold or withdraw life support”	Antimicrobials were withheld or discontinued in 74 patients.	N/A
Jakobson et al.^23^ (2004, Israel, Europe)	High	Ecological study	To evaluate changes over time in frequency and types of forgoing LST in ICU patients.	Patients who died and/or had forgoing of LST in the ICU (N=77)	Decision to forgo LST.	Antimicrobials were never withheld or stopped except for medical indications.	N/A
Datta et al.^24^ (2020, USA, North America)	High	Cohort study	To evaluate the relationship between antibiotic use and hospital stay in older adults with advanced cancer.	Older adults with advanced cancer transitioned to comfort measures in the ICU (N=276)	Transition to comfort measures during admission.	Seventy-seven patients who received antimicrobials died in the ICU.	27.9% (77/276)
Huang et al.^25^ (2010, Chinese-Taipei, Asia-Pacific)	Middle	Descriptive epidemiological study	To survey DNR factors and evaluate the impact on treatment in the surgical ICU.	Patients with DNR consent in the surgical ICU (N=601)	DNR consent.	A total of 463 patients used antimicrobials at time of death.	77.0% (463/601)
Chen et al.^26^ (2014, Chinese-Taipei, Asia-Pacific)	Middle	Cohort study	To examine care for patients with two DNR order protocols. DNR Comfort Care: only comfort care after DNR order; DNR Comfort Care-Arrest: aggressive interventions until arrest.	Patients with a DNR comfort care order in the ICU (N=88)	DNR comfort care	Sixty-six patients received antimicrobials before DNR comfort care, and 8 patients received antimicrobials after DNR comfort care.	9.1% (8/88)
Kim et al.^27^ (2022, Korea, Asia-Pacific)	High	Descriptive epidemiological study	To assess antimicrobial prescribing and factors limiting use in terminally ill patients with LST suspension.	Patients who died within 7 days after implementation of suspended LST (N=426)	Suspension of LST in terminally ill patients.	Antimicrobials were streamlined (de-escalation) in 123 patients after suspension of LST.	N/A

**Table 1:** (continued)

Authors (Year, country, region)	Income level	Study type	Primary aim of the study	Study population (N)	EOL definition	Outcomes of antimicrobial use in EOL care in ICU	Proportion of antimicrobial use
Psirides and Sturland^28^ (2009, New Zealand, Asia-Pacific)	High	Descriptive epidemiological study	To assess withdrawal methods of active treatment in ICU patients and compare with staff beliefs.	Patients who had active treatment withdrawn in the ICU (N=40)	Active treatment withdrawal.	Three patients received antimicrobials during the withdrawal process.	7.5% (3/40)
Yazigi et al.^29^ (2005, Lebanon, Asia-Pacific)	Middle	Descriptive epidemiological study	To evaluate withholding and withdrawing LST decisions in ICU patients.	ICU patients with withholding or withdrawal of LST in the ICU (N=43)	LST withholding and withdrawal.	Antimicrobial therapy was withheld or withdrawn in 8 patients.	N/A
Lesieur et al.^30^ (2015, France, Asia-Pacific)	High	Descriptive epidemiological study	To investigate the incidence of withholding or withdrawal decisions in French ICUs and evaluate their implementation according to legislation.	Patients with withholding or withdrawal of care who died in the ICU (N=777)	Withholding or withdrawal of procedures according to the French Leonetti law.	In total, 152 patients discontinued antibiotics.	N/A
Lesieur et al.^31^ (2018, France, Europe)	High	Ecological study	To compare withholding or withdrawing practices between 2012 and 2016.	Patients with withholding or withdrawal of procedures in the ICU (N=264)	Withholding or withdrawal of procedures.	In total, 179 patients continued antibiotics, and 17 patients discontinued antibiotics.	N/A
Wakatake et al.^32^ (2022, Japan, Asia-Pacific)	High	Descriptive epidemiological study	To evaluate the clinical courses of patients with severe brain damage and assess EOL care preferences in Japanese hospitals.	Patients with severe brain damage and poor neurological prognosis who had withdrawal or withholding of LST (N=475)	Withdrawal or withholding of LST.	In total, 101 patients continued antimicrobials, and 34 patients received new antimicrobials. No patients discontinued antimicrobials.	28.4% (135/475)
Kranidiotis et al.^33^ (2010, Greece, Europe)	High	Cohort study	To examine the frequency, type, and rationale for limiting LST in Greek ICUs, including family involvement.	Patients who died in the ICU (N=182)	Limitation of LST.	Two patients withdrew antimicrobials.	N/A
Chen et al.^34^ (2017, USA, North America)	High	Descriptive epidemiological study	To review current EOL care practice patterns among U.S. cystic fibrosis care centers.	Patients who died in cystic fibrosis care centers within 7 days before death (N=96)	Last week of life in cystic fibrosis care centers.	Eighty-five patients used intravenous antimicrobials in the 7 days preceding death.	88.5% (85/96)
Urvay and Karagöz^35^ (2021, Turkey, Europe)	Middle	Descriptive epidemiological study	To investigate antibiotic use in patients with terminal-stage cancer undergoing palliative care who died during hospitalization.	Patients with terminal-stage cancer undergoing palliative care who died in-hospital (N=57)	Defined by inability to receive curative treatments, receiving palliative care only for symptoms.	Sixteen patients discontinued antimicrobial therapy.	5.3% (3/57)
Buckley et al.^36^ (2004, China, Asia-Pacific)	Middle	Descriptive epidemiological study	To examine the frequency and processes for limiting LST (withdrawal/withholding) in critically ill ICU patients in China.	Patients who died in the ICU with limitation of LST (N=288)	Limitation of LST, including treatment for infection, hemodynamic instability, poor tissue oxygenation, or biochemical or hematologic derangements.	Antimicrobials were withheld in 3 patients. No patients discontinued antimicrobial therapy.	N/A
Bastos et al.^37^ (2023, Brazil, Latin America)	Middle	Descriptive epidemiological study	To evaluate reduction in antimicrobial use through time-reduction strategy in palliative ICU care.	ICU patients under exclusive palliative care monitored by an ICU pharmacist (N=8)	Exclusive palliative care.	Association was found between reduction of antimicrobial therapy and exclusive palliative care.	N/A
Leong and Tai^38^ (2001, UK, Europe)	High	Case-control study	To study the practice of foregoing LST between young-old and old-old individuals.	Patients foregoing LST in medical ICUs (N=57)	Foregoing LST included both withdrawal of and withholding of care.	Antimicrobial therapy was foregone in 2 patients.	N/A
Nolin and Andersson^39^ (2003, Sweden, Europe)	High	Descriptive epidemiological study	To analyze the frequency of withdrawal decisions, grounds for discontinuation of LST, and outcomes in ICU patients.	Patients with LST withdrawal in ICU (N=318)	Withdrawal of LST.	Antimicrobials were discontinued in 216 patients.	N/A

Abbreviations: ICU, intensive care unit; EOL, end-of-life; LST, life-sustaining treatment; DNR, do-not-resuscitate; N/A, not available.

**Table 2:** Risk of bias assessment using NOS score for 7 cohort studies and 1 case-control study

Cohort studies					
Authors	Selection	Comparability	Outcome	Total Score	AHRQ Standard
Merel et al.^14^	★★★☆	☆☆	★★★	6	Poor
Choudhuri et al.^16^	★★★☆	☆☆	★★★	6	Poor
Alonso et al.^20^	★★★☆	☆☆	★★★	6	Poor
Mani et al.^21^	★★★☆	☆☆	★★★	6	Poor
Datta et al.^24^	★★★☆	☆☆	★★★	6	Poor
Chen et al.^26^	★★★★	★★	★★★	9	Good
Kranidiotis et al.^33^	★★★☆	☆☆	★★★	6	Poor
Case-control study					
Authors	Selection	Comparability	Exporsure	Total Score	Quality
Leong and Tai et al.^38^	★★★★	☆☆	★★★	7	Poor

NOS, Newcastle–Ottawa Scale; AHRQ, Agency for Healthcare Research and Quality
