# Supplementary material for: Antimicrobial use in patients with end-of-life status in intensive care units: A systematic review and meta-analysis
Source: Fujita Med J. 2025 Nov 5;12(1):20–8. doi: 10.20407/fmj.2025-020 (PMC12865281; doi:10.20407/fmj.2025-020)
Supplement: Supplementary file 1 — Supplementary Table [file fmj-12-020-s001.pdf]

Supplementary Table: Search strategy

Database: Medline (PubMed) – Search Date: 08172023

#1	"Anti-Bacterial Agents"[Mesh] OR "Anti-Bacterial Agents" [Pharmacological Action] OR "Bacterial Infections/drug therapy"[Mesh]
#2	"Anti bacterial*"[tiab] OR "Antibacterial*"[tiab] OR "Antimicrob*"[tiab] OR "Antibiotic*"[tiab] OR "Bacterial infect*"[tiab] OR "Microbicides"[tiab]
#3	("life support"[tiab] OR "limit*"[tiab] OR "withhol*"[tiab] OR "withdraw*"[tiab] OR "withheld"[tiab]) AND ("treatment"[tiab] OR "therapy"[tiab])
#4	#1 OR #2 OR #3
#5	"Terminal Care"[Mesh] OR "Hospice Care"[Mesh] OR "Terminally Ill"[Mesh] OR "Palliative Care"[Mesh] OR "Life Support Care"[Mesh] OR "Withholding Treatment"[Mesh]
#6	"End of life"[tiab] OR "Terminal care"[tiab] OR "Dying"[tiab] OR "Terminally ill"[tiab] OR "Palliative care"[tiab] OR "Resuscitation order*"[tiab] OR "Critical illness"[tiab] OR "Life support"[tiab] OR "Withhold*"[tiab] OR "Withdraw*"[tiab] OR "End stage"[tiab] OR "terminal*"[tiab] OR "last days"[tiab] OR "palliative"[tiab] OR "DNR" [tiab] OR "comfort care"[tiab] OR "terminal illness"[tiab] OR "hospice" [tiab] OR "advanced disease"[tiab] OR "advanced illness"[tiab] OR "do-not-resuscitate"[tiab]
#7	#5 OR #6
#8	"Intensive Care Units"[Mesh] OR "Critical Care"[Mesh]
#9	"Intensive care unit*"[tiab] OR "Critical care"[tiab] OR "Intensive care"[tiab] OR "Emergen*"[tiab]
#10	#8 OR #9

#11	"Review" [Publication Type] OR "Comment" [Publication Type] OR "Editorial" [Publication Type] OR "Systematic Review" [Publication Type] OR "Meta-Analysis" [Publication Type] OR "Letter" [Publication Type] OR "Case Reports" [Publication Type] OR "Guideline" [Publication Type]
#12	#4 AND #7 AND #10 NOT #11

Database: Embase – Search Date: 08172023

#1	'antibiotic agent'/exp OR 'antimicrobial therapy'/exp OR 'antibacterial activity'/exp OR 'bacterial infection'/exp/dm_dt
#2	'terminal care'/exp OR 'hospice care'/exp OR 'palliative therapy'/exp OR 'terminally ill patient'/exp OR 'life sustaining treatment'/exp OR 'treatment withdrawal'/exp
#3	'intensive care unit'/exp OR 'intensive care'/exp
#4	#1 AND #2 AND #3
#5	'anti bacterial*':ab,ti OR 'antibacterial*':ab,ti OR 'antimicrob*':ab,ti OR 'antibiotic*':ab,ti OR 'bacterial infect*':ab,ti OR 'microbicides':ab,ti
#6	('life support':ab,ti OR 'limit*':ab,ti OR 'withhol*':ab,ti OR 'withdraw*':ab,ti OR 'withheld':ab,ti) AND ('treatment':ab,ti OR 'therapy':ab,ti)
#7	#5 OR #6
#8	'end of life':ab,ti OR 'terminal care':ab,ti OR 'dying':ab,ti OR 'terminally ill':ab,ti OR 'palliative care':ab,ti OR 'resuscitation order*':ab,ti OR 'critical illness':ab,ti OR 'life support':ab,ti OR 'withhold*':ab,ti OR 'withdraw*':ab,ti OR 'end stage':ab,ti OR 'terminal*':ab,ti OR 'last days':ab,ti OR 'palliative':ab,ti OR 'DNR':ab,ti OR 'comfort care':ab,ti OR 'terminal illness':ab,ti OR 'hospice':ab,ti OR 'advanced disease':ab,ti OR 'advanced illness':ab,ti OR 'do-not-resuscitate':ab,ti

#9	'intensive care*':ab,ti OR 'critical care':ab,ti OR 'emergen*':ab,ti
#10	#7 AND #8 AND #9
#11	'note'/it OR 'editorial'/it OR 'letter'/it OR 'conference abstract'/it OR 'case report'/de OR 'animal experiment'/de OR 'nonhuman'/de
#12	#4 OR #10 NOT #11
